# Design of Nanotechnological Carriers for Ocular Delivery of Mangiferin: Preformulation Study

**DOI:** 10.3390/molecules27041328

**Published:** 2022-02-16

**Authors:** Debora Santonocito, Maria Vivero-Lopez, Maria Rosaria Lauro, Cristina Torrisi, Francesco Castelli, Maria Grazia Sarpietro, Carmelo Puglia

**Affiliations:** 1 Department of Drug and Health Sciences, University of Catania, Viale Andrea Doria n 6, 95125 Catania, Italy; cristina.torrisi@phd.unict.it (C.T.); fcastelli@unict.it (F.C.); mg.sarpietro@unict.it (M.G.S.); capuglia@unict.it (C.P.); 2 Departamento de Farmacología, Farmacia y Tecnología Farmacéutica, I+D Farma (GI-1645), Facultad de Farmacia and Health Research Institute of Santiago de Compostela (IDIS), Universidade de Santiago de Compostela, 15782 Santiago de Compostela, Spain; mariavivero.lopez@usc.es; 3 Department of Pharmacy, University of Salerno, Via Giovanni Paolo II, 84084 Fisciano, Italy; lauro@unisa.it; 4 NANO-i—Research Centre on Ocular Nanotechnology, University of Catania, 95125 Catania, Italy

**Keywords:** mangiferin, nanostructured lipid carriers, ocular drug delivery, eye diseases

## Abstract

(1) Background: Mangiferin (MGN) is a natural compound, showing anti-inflammatory and antioxidant activities for the potential treatment of eye diseases. The poor physicochemical features of MGN (low solubility and high instability) justify its nanoencapsulation into nanostructured lipid carriers (NLC) to improve its ocular bioavailability. (2) Methods: Firstly, MGN-NLC were prepared by the high shear homogenization coupled with the ultrasound (HSH−US) method. Finally, unloaded and MGN-loaded NLC were analyzed in terms of ocular tolerance. (3) Results: MGN-NLC showed good technological parameters suitable for ocular administration (particle size below 200 nm). The ORAC assay was performed to quantify the antioxidant activity of MGN, showing that the antioxidant activity of MGN-NLC (6494 ± 186 μM TE/g) was higher than that of the free compound (3521 ± 271 μM TE/g). This confirmed that the encapsulation of the drug was able to preserve and increase its activity. In ovo studies (HET-CAM) revealed that the formulation can be considered nonirritant. (4) Conclusions: Therefore, NLC systems are a promising approach for the ocular delivery of MGN.

## 1. Introduction

In recent years, intense research has been conducted to treat posterior ocular segment diseases using topical formulations able to overcome the complex anatomy of the eye [1]. The use of conventional eye drops is limited by precorneal drug removal mechanisms and physiological barriers [2,3] that oppose drug penetration, limiting the ocular bioavailability to 5–10% [4]. This means that the achievement of therapeutic effects requires frequent instillations, with consequent low patient compliance [5]. Other strategies are the use of ocular injections (peribulbar, retrobulbar and subconjunctival) and the application of ocular implants; however, both therapies show drawbacks due to high costs [6]. 

The advent of nanotechnology has made important innovations in the field of ophthalmology [7]. Over the years, two generations of lipid nanoparticles have been developed: the first generation consists of solid lipid nanoparticles (SLN) and the second generation consists of nanostructured lipid carriers (NLC). Drug encapsulation into these nanocarriers has been reported to increase drug solubility, stability, penetration, targeted cell uptake, tolerability and interaction with the ocular mucosa [8,9,10,11,12]. NLC present numerous advantages, such as controlled drug release, high drug loading and excellent tolerability [4,13]. NLC are made up of a mixture of lipid solid and liquid (oil), stabilized by a surfactant. The oily fraction is responsible for a distortion of the lipid crystals; this particular internal structure allows a high loading capacity and a remarkable physical stability. Therefore, NLC are currently studied as delivery systems for the treatment of the most important ocular disorders affecting the posterior eye segment [4,5,6,7,8,9,10,11,12,13,14,15,16].

**Figure 1:**
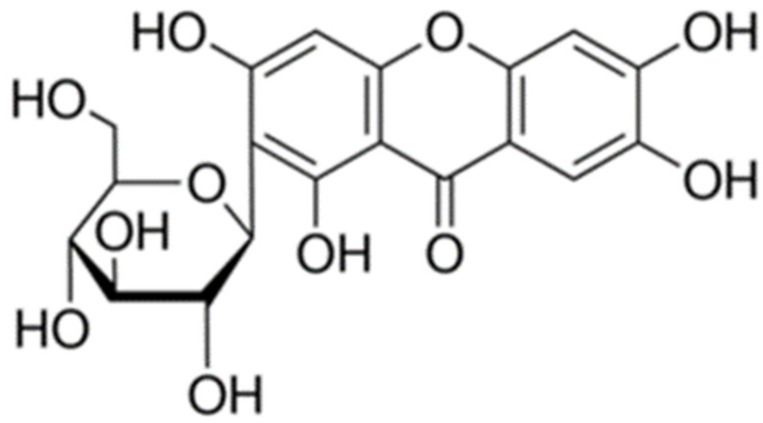
Chemical structure of mangiferin (MGN).

Many eye diseases are related to oxidative stress, such as macular degeneration and diabetic retinopathy [17] as overproduction of reactive oxygen species (ROS) affects neurons and retinal vessels. Thus, the attention on antioxidants has been greatly increased in the field of ophthalmology. Ocular applications of antioxidants are hindered by stability and bioavailability problems, which can be overcome by encapsulation into nanocarriers [18]. Several studies pointed to mangiferin (MGN) for the potential treatment of eye diseases [19,20]. MGN (2-b-D-glucopyranosyl-1,3,6,7-tetrahydroxyxanthone) is a polyphenol compound (Figure 1), primarily extracted from the leaves, stem barks and fruits of *Mangifera indica* L., exhibiting a variety of therapeutic effects, of which a strong antioxidant activity [21,22,23,24]. 

To overcome the stability and low aqueous solubility (0.111 mg/mL) limitations [25,26,27], the feasibility of encapsulating MGN in NLC was investigated in a previous paper [20]. In that work, NLC were obtained combining glyceryl monosterate, Gelucire 44/14, Miglyol 812, Labrasol and large proportions of Tween 80 surfactant, but the antioxidant activity of MGN was not investigated. Other nanotechnological systems have been developed in order to increase the solubility and bioavailability of MGN, such as self-assembled phytosomal soft nanoparticles encapsulated with a phospholipid complex (MPLC SNPs) [28] and polymeric nanoparticles [29,30], demonstrating the importance of the nanoencapsulation strategy.

The aim of this preliminary study was to design NLC for MGN using more biocompatible and safer surfactant and lipids and to elucidate their suitability for the potential treatment of posterior eye segment disorders using a variety of in vitro and in ovo approaches. Thus, Miglyol 812 was mixed with Compritol 888 ATO (COMP), a mixture of mono-, di- and triglycerides of behenic acid, which shows excellent regulatory and safety profiles [31]. Moreover, Lutrol F68 was used as a surfactant at a very low proportion (0.4% *w*/*v*). MGN-NLC were prepared by the high shear homogenization coupled with the ultrasound (HSH-US) method. MGN-NLC were characterized for their mean size, zeta-potential, size distribution and morphology. The mechanism by which NLC interact with a biomembrane model of multilamellar vesicles (MLV) was investigated using differential scanning calorimetry (DSC) [32] while the antioxidant activity of MGN was evaluated by the ORAC assay. Finally, the obtained formulations were analyzed in terms of ocular tolerance. Therefore, MGN-NLC are a promising approach for treating retinal diseases as they are able to bypass the problems of MGN (low water solubility and instability) and administer it in the form of eye drops.

## 2. Results

### 2.1. NLC Preparation and Characterization

MGN-NLC showed a mean diameter of 148.9 ± 0.1 nm, a PDI value around 0.21 ± 0.02 and a zeta potential value of −23.5 mV (Table 1), predicting a good long-term stability for the formulation [33]. The encapsulation efficiency, evaluated by UV/VIS spectrometry, was about 92%. The drug loading was about 4.7%.

### 2.2. Transmission Electron Microscopy (TEM)

**Figure 2:**
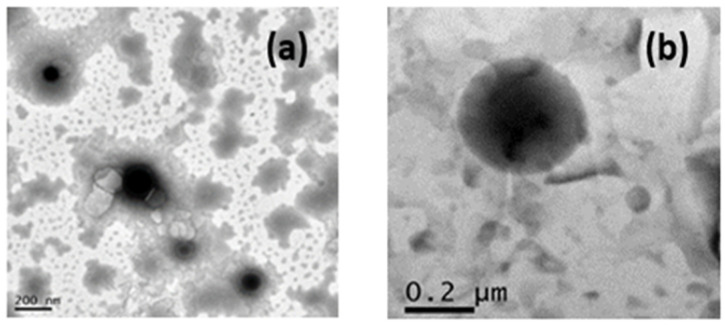
(**a**,**b**) Transmission electron microscopy images of MGN-NLC. The scale bar represents 200 nm.

The morphology of the MGN-NLC was performed using TEM (Figure 2). In agreement with the DLS data, the TEM images showed that the nanoparticles are suitable for ocular administration (particle size below 200 nm) [6,34,35,36] and are well structured.

### 2.3. DSC Analysis

#### 2.3.1. NLC and MLV Analysis

**Figure 3:**
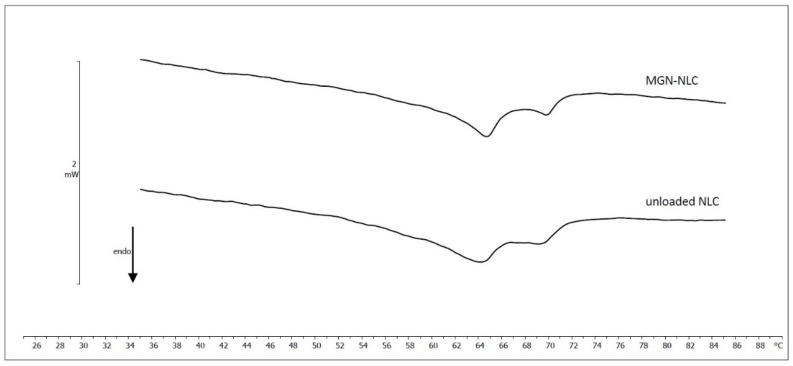
Calorimetric curves, in heating mode, of unloaded NLC and MGN-NLC.

**Figure 4:**
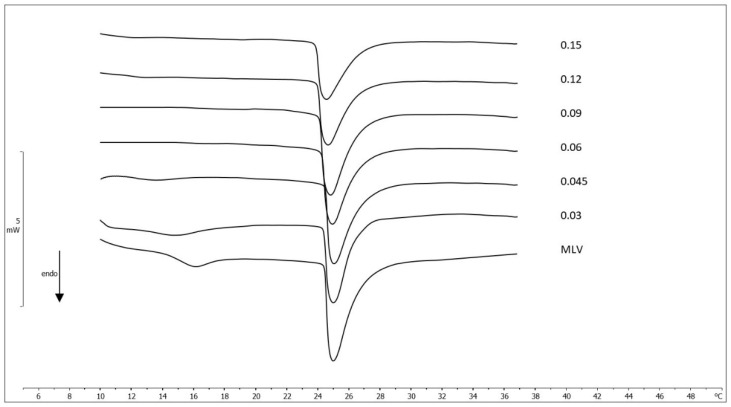
Calorimetric curves, in heating mode, of MLV containing a different molar fraction of MGN.

The thermotropic behavior of unloaded NLC and MGN-NLC was evaluated by DSC. The calorimetric curves of the single components are shown in Figure S1 of the supporting information. Compritol, the solid lipid used to obtain NLC, showed a calorimetric peak centered at 71.57 °C, with an enthalpy variation of −124.76 J/g. The calorimetric curve of Lutrol was characterized by a peak centered at 52.49 °C, with an enthalpy variation of −122.93 J/g; Mygliol did not show any calorimetric peak but only a flat line. The calorimetric curves of NLC and MGN-NLC are shown in Figure 3. Unloaded NLC were characterized by a main peak centered at 64.24 °C and a shoulder at a higher temperature as well as an enthalpy variation of −66.13 J/g of Compritol. The NLC melting temperature was about 7 °C lower than that of Compritol, owing to an increase of surface area resulting from NLC colloidal sizes and from interactions between the solid lipid and MIG and surfactant molecules that led to a less ordered structure. The MGN-NLC run had a main peak at 64.68 °C and a smaller one at 69.68 °C as well as an enthalpy variation of −65.08 J/g of Compritol. These results suggest that MGN affected the thermotropic behavior of the nanocarriers and, therefore, may interact with lipid constituents of the NLC formulation. The DSC analysis of MGN was also carried out. In accord with the literature data [37,38], MGN had an endothermic peak centered at 272.11 °C (Figure S1, supporting information). The effect of different molar fraction of MGN (0.03; 0.045; 0.06; 0.09; 0.12; 0.15) on the biomembrane model made of MLV of 1,2-dimyristoyl-sn-glycero-3-phosphocholine (DMPC) was also investigated by DSC (Figure 4) [31,39,40]. All recorded calorimetric curves were referred to those made of pure DMPC MLV. The latter one showed a pretransition peak at about 16 °C, related to the transition from the gel (ordered) to the ripple phase, and a main peak at 25 °C, due to the transition from the ripple to the liquid-crystalline (disordered) phase. Any foreign molecules in the lipid bilayer may act as impurities and cause variations of the thermodynamic parameters (T_m_ and ∆H). In the case of MGN, the pretransition peak decreased and finally disappeared as the amount of molar fraction increased while the main peak slightly shifted to a lower temperature, and its enthalpy variation remained almost unchanged. This result indicated that MGN exerted only a low effect on DMPC MLV.

#### 2.3.2. MLV/MGN Kinetic Experiments

**Figure 5:**
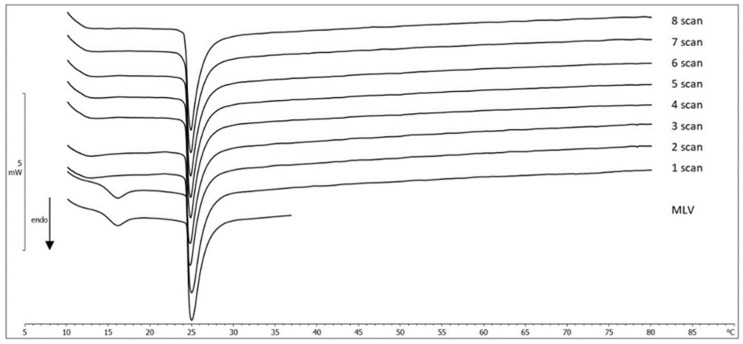
Calorimetric curves, in heating mode, of MLV left in contact with the solid MGN at increasing times.

The ability of the MGN to be absorbed by the phospholipid membranes through an aqueous medium was studied by leaving an amount of the DMPC MLV dispersion in contact with a defined quantity of powdered compound. If the compound dissolves through the aqueous medium and is able to interact with the phospholipid bilayers, it is possible to observe a gradual variation of the calorimetric curve shape of DMPC MLV (reported as a reference). In this case, in all recorded calorimetric curves, the pretransition peak disappeared whereas the main peak did not show any variation (Figure 5). Therefore, MGN cannot be incorporated in the MLV structure, probably due to its low water solubility. 

#### 2.3.3. MLV/NLC Kinetic Experiments

**Figure 6:**
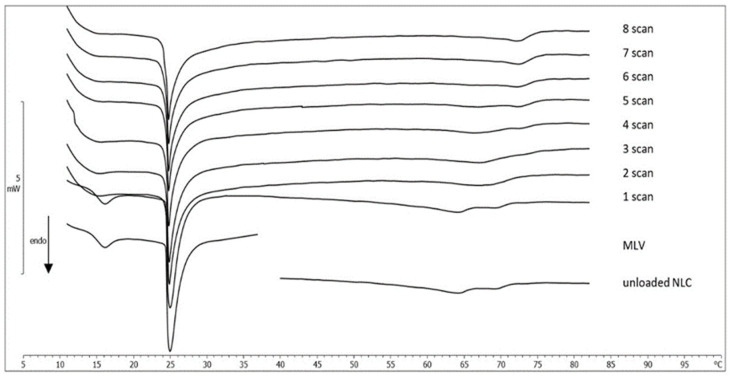
Calorimetric curves, in heating mode, of MLV left in contact with unloaded NLC at increasing times.

**Figure 7:**
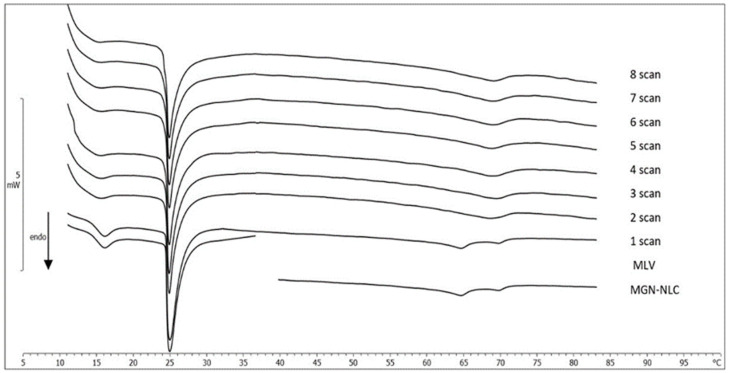
Calorimetric curves, in heating mode, of MLV left in contact with MGN-NLC at increasing times.

The interaction between DMPC MLV, used as biological membrane model, and NLC (unloaded NLC and MGN-NLC) was evaluated by DSC. An amount of DMPC MLV and NLC were put in contact, and the kinetic experiments were carried out. The calorimetric curves, recorded at one-hour intervals, were compared with the individual curves of samples (Figure 6 and Figure 7, respectively). Regarding the first experiment, the pretransition peak of DMPC MLV gradually vanished while the main peak underwent variations in height and width, and the enthalpy variation gradually decreased (Table 2). Firstly, the calorimetric curve of NLC showed a main peak and a shoulder, characteristic of unloaded NLC; then, as the contact time increased, the peak merged with the shoulder, giving a unique peak at a higher temperature. This is clear evidence of the interaction between MLV and unloaded NLC; in particular, DMPC MLV maintained their structure while the NLC structure underwent marked variations. A similar behavior can be seen in the DMPC MLV and MGN-NLC interactions. DMPC MLV lost the pretransition peak, and the main peak showed a reduction, associated with a gradual decrease of the enthalpy variation (Table 2). The shape of the MGN-NLC calorimetric curve, characterized by a well-defined two peaks structure, did not initially change, but the two peaks gradually merged in a unique peak that, in the last scans, was centered at 69.11 °C and remained stable. Therefore, there was an interaction between MLV and MGN-NLC. In order to confirm that the variations of the unloaded NLC and MGN-NLC were due to the interaction with MLV, simple experiments were carried out. Unloaded NLC and MGN-NLC were separately submitted to the same calorimetric conditions of the kinetic experiments. The calorimetric curves did not show any variation over the time (data not shown), confirming the interaction between NLC and MLV. We can hypothesize that NLC can enter the MLV structure and release MGN.

### 2.4. ORAC Assay

During the ORAC assay, the decay of the fluorescence of fluorescein was monitored. A calibration curve was obtained by plotting the area under the curve (AUC) against a Trolox concentration (r^2^ = 0.9913). The ORAC assay was applied to determine the antioxidant activity of the MGN-NLC (0.002 M) using unloaded NLC as a control and a MGN solution (free compound, 0.002 M), reaching values of 6494 ± 186, 769 ± 52 and 3521 ± 271 μM TE/g, respectively (Table 3). The obtained values showed that the antioxidant activity of MGN-NLC was higher than that of the free compound (MGN solution). 

### 2.5. HET−CAM Assay

**Figure 8:**
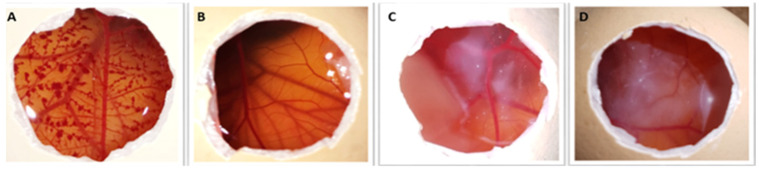
Pictures of chorioallantoic membrane (CAM) after the application of (**A**) 0.1 M NaOH so-lution (positive control), (**B**) 0.9 wt% NaCl solution (negative control), (**C**) unloaded NLC and (**D**) MGN-NLC.

HET−CAM is an in ovo assay highly sensitive for predicting the ocular irritation effect [41]. Potential ocular irritation effects of MGN-NLC and unloaded NLC were determined on the chorioallantoic membrane (CAM) of fertilized hen eggs. No damage to the blood vessels on the CAM surface after a 5 min period of contact with MGN-NLC was detected, as reported in Figure 8. The IS was 0.0, as occurred with the negative control (0.9% NaCl). Thus, MGN-NLC can be classified as nonirritant (IS < 1) since no hemorrhage, lysis or coagulation were observed. The slight white halo observed in Figure 8C,D was due to the milky appearance of the tested formulations.

### 2.6. Hemolysis Assay

Investigation of the hemocompatibility profiles of tested formulations revealed that they had hemolysis effects of about 10%. The percentage of hemolysis for unloaded NLC and MGN-NLC were 11.16 ± 3.12% and 8.10 ± 0.95%, respectively. 

## 3. Discussion

MGN-loaded NLC were formulated by high shear homogenization coupled with an ultrasound (HSH−US) method using Compritol^®^ 888 ATO and Miglyol 812^®^ as the lipid phase and Lutrol F68^®^ (Poloxamer 188) as the surfactant. The importance of this combination of HSH-US in controlling the size of lipid nanoparticles has been demonstrated [42]. Therefore, this method proved to be valid, highly reproducible and suitable for the formulation of ocular nanocarriers. DLS data showed good technological parameters, with a size below 200 nm (as confirmed by TEM images), a PDI around 0.2 and a zeta potential value approximately −20 mV, predicting a good long-term stability. This could be due to the presence of Poloxamer on the particle surface, thereby creating a stabilizer layer [43,44,45]. The choice of surfactant is very important because it controls the particle size and the stability, preventing their aggregation during storage [46,47]. In a previous report NLC prepared, combining glyceryl monosterate, Gelucire 44/14, Miglyol 812, Labrasol and large proportions of Tween 80 surfactant [20], there was a smaller size (50–70 nm), similar PDI and larger zeta potential (−30 to −36 mV).

Calorimetric data demonstrated that MGN affects the thermotropic behavior of NLC; in fact, the one peak and one shoulder curve of unloaded NLC became a two peaks curve in MGN-NLC, demonstrating that MGN was incorporated in NLC. The kinetic experiments, carried out to evaluate an interaction between MLV and unloaded NLC and between MLV and MGN-NLC, showed the variation both of the MLV as well as of the unloaded NLC and MGN-NLC peaks, indicating that unloaded NLC and MGN-NLC interacted with MLV [48]. The data were confirmed by the absence of variations in the calorimetric curves of unloaded NLC and MGN-NLC submitted to the same experimental conditions.

The antioxidant activity of MGN-loaded NLC was determined by the ORAC assay using unloaded NLC as a control MGN-NLC and an MGN solution (free compound); the concentration of MGN was 0.002 M in both samples. The values obtained showed that the antioxidant activity of MGN-NLC (6494 ± 186 μM TE/g) was higher than that of the free compound (3521 ± 271 μM TE/g). This confirmed that the encapsulation of the drug was able to preserve and increase its activity [49]. The antioxidant activity against radicals ranked in the order MGN-NLC > MGN solution > unloaded NLC (control).

In terms of safety, all the components used for the formulation of nanoparticles are safe [13,45,50,51] as confirmed by HET−CAM test. This test is an alternative to the Draize test and provides information on the potential ocular irritancy of the formulations through the irritation score (IS). CAM is a vascular tissue that responds to harmful agents with a complete inflammatory process (hemorrhage, lysis or coagulation). The IS value was 0.0, which occurred with the negative control (0.9% NaCl), while the IS of the positive control was about 18.82. No damage to the blood vessels on the CAM surface after a 5 min period of contact with MGN-NLC was observed; therefore, it can be qualified as a nonirritating formulation. Moreover, MGN-NLC showed adequate hemocompatibility as the unloaded NLC.

The promising findings of the present work suggest future research directions to test in vivo the efficacy of MGN-NLC in the management of retinal diseases involving a deficit of antioxidant defenses (i.e., macular degeneration).

## 4. Materials and Methods

### 4.1. Materials

Solid lipid Compritol 888 ATO (COMP, a mixture of mono-, di-, and triglycerides of behenic acid) was purchased from Gattefossè (Milan, Italy); oil Miglyol 812 (MIG, a mixture of medium-chain triglycerides) was obtained from Eigenmann & Veronelli S.p.A. (Milan, Italy) and Lutrol F68 (MW 8400 g/mol) was provided by BASF ChemTrade GmbH (Burgbernheim, Germany). Mangiferin (MGN, MW 422.33 g/mol), Trolox (MW 250.29 g/mol) and 2,2’-azobis(2-methylpropionamidine) dihydrochloride (AAPH, MW 271.19 g/mol) were obtained from Sigma-Aldrich (St. Louis, MO, USA). Fluorescein disodium salt (MW 332.31 g/mol) was purchased from Acros Organics (Milan, Italy). Triton X-100, propylene glycol and PBS commercial (10×) were purchased from Sigma-Aldrich (Madrid, Spain). Ultrapure water (18.2 Momega) was obtained by filtering through millipore (MilliQ^®^, Millipore Ibérica, Madrid, Spain).

### 4.2. MGN-NLC Preparation

MGN-NLC were prepared by high shear homogenization coupled with an ultrasound (HSH−US) method [52] that was slightly modified. Briefly, the lipid phase containing MGN (0.1% *w*/*v*), COMP (0.6 g) and MIG (0.4 g) were first melted at 80 °C then uniformly mixed in the surfactant solution at the same temperature (Lutrol F68 0.4% *w*/*v*) by using a high-speed stirrer (Ultra-Turrax T25, IKA-Werke GmbH &Co. Kg, Staufen, Germany) for 10 min. The obtained preemulsion was ultrasonified by a Labsonic 2000 (B. Braun, Melsunen, Germany) for 8 min. The hot dispersion was then cooled by dilution in 25 mL of additional water at 4 °C. Unloaded NLC was prepared by the same procedure without adding MGN.

### 4.3. MGN-NLC Physical Characterization

The average size (Z-Ave), polydispersity index (PDI) and the zeta potential (ZP, ξ) of MGN-NLC were assessed by dynamic light scattering (DLS) using a Zeta Sizer Nano-ZS90 (Malvern Instrument Ltd., Worcs, Malvern, UK). Experiments were carried out at 20 ± 0.2 °C and at a scattering angle of 90°. Samples (100 μL of NLC suspension) were diluted with distilled water (900 μL). Each formulation was measured at least in triplicate. 

### 4.4. Transmission Electron Microscopy (TEM)

MGN-NLC morphology was investigated using TEM (JEOL JEM-101). The formulation was diluted 100 times with water and then deposited on the surface of a 200 mesh Formvar^®^-coated copper grid (TAAB Laboratories Equipment, Ltd., Aldermaston, UK). The acceleration voltage was set to 190 kV. After drying, the specimen was covered with chromium prior to imaging (Quorum Q150T ES East Grinstead, West Sussex, UK).

### 4.5. Encapsulation Efficiency and Drug Loading

NLC formulation was diluted in H_2_O, filtered through a 0.22 μm sterile syringe filter (TS-900-045-S, Test Scientific, Perugia, Italy) and freeze dried overnight. A defined amount of the lyophilized filtrate was solubilized in methanol, and the MGN content was measured by UV spectrophotometry at 257 nm (T80^+^ UV/VIS Spectrometer, PG Instrument Ltd., Lutterworth, UK). Calibration curves of MGN were performed on six different concentrations (range 0.5–10 μg/mL). Each point was the average of three measurements. The entrapment efficiency (EE) of MGN in the NLC was calculated from Equation (1):

EE% = [(mgMGN_total_ − mgMGN_free_)/mgMGN_total_] × 100 (1)

The drug loading (DL) was calculated from Equation (2):

DL% = [(mgMGN_total_ − mgMGN_free_)/weight of lipids] × 100 (2)

### 4.6. DMPC/MGN MLV Preparation

DMPC/MGN MLV were prepared as follows. DMPC was dissolved in chloroform:methanol (1:1 *v*/*v*), and MGN was dissolved in methanol. Aliquots of a DMPC solution (0.010325 mmol) were delivered in glass vials, and aliquots of the MGN solution were added to have defined molar fractions of MGN with respect to DMPC (0.00, 0.03, 0.045, 0.06, 0.09, 0.12 and 0.15). The solvents were evaporated under a nitrogen flow at 37 °C. The obtained phospholipid films were freeze dried overnight to eliminate any solvent traces, and 50 mM of a Tris buffer solution at pH 7.4 was added to the films to reach 0.0614 mmoles DMPC/mL. The dispersions were kept at 37 °C for 1 min, vortexed three times for 1 min and then kept at 37 °C for 60 min.

### 4.7. Differential Scanning Calorimetry (DSC)

The DSC studies were carried out with a Mettler Toledo STAR^e^ system equipped with a DSC-822^e^ calorimetric cell and the Mettler TA-STAR^e^ software. The formulation was poured in aluminum (160 µL), which was sealed after filling. The reference pan contained a Tris buffer solution (50 mM). The maximum sensitivity obtainable by the calorimetric system was used. The procedure of the Mettler TA-STAR^e^ software was followed for the calibration of temperature and enthalpy changes; indium, stearic acid and cyclohexane were used.

#### 4.7.1. MLV and NLC Analysis

MLV (120 µL; 0.007375 mmoles of DMPC) were placed into the calorimetric pan and subjected to DSC heating and cooling scans: (1) a scan from 5 to 37 °C (2 °C/min) and (2) a scan from 37 to 5 °C (4 °C/min), both conducted at least three times to check the results’ reproducibility. NLC (120 µL) were put into the calorimetric pan and subjected to the DSC analysis as follows: (1) a heating scan from 25 to 85 °C at a rate of 2 °C/min and then (2) a cooling scan from 85 to 5 °C at a rate of 4 °C/min; each scan was conducted at least three times to check the reproducibility of the results.

#### 4.7.2. Kinetic Experiments

##### Interaction between MLV and MGN

An amount of MGN corresponding to 0.5 molar fraction with respect to DMPC was weighed in the calorimetric pan, and 120 µL of a MLV sample was added. Then, the pan was hermetically sealed, and the interaction was monitored by the DSC analysis as follows: (i) a heating scan at a rate of 2 °C/min between 5 and 85 °C, (ii) a cooling scan at a rate of 4 °C/min between 85 and 37 °C, (iii) an isothermal period (1 h) at 37 °C to allow the NLC to interact with and permeate the phospholipid bilayers of MLV and (iv) a cooling scan between 37 and 5 °C at a rate of 4 °C/min. This procedure was run at least eight times to follow eventual variations in the behavior of the MLV made with DMPC (DMPC MLV) and of NLC due to the time-dependent interactions. Each experiment was repeated three times.

##### Interaction between MLV and NLC

Thirty microlitres of MLV (0.245 mmoles DMPC/mL) were placed in the calorimetric pan, followed by the addition of 90 µL of NLC; the pan was hermetically sealed, and the interaction was monitored as described above.

### 4.8. Antioxidant Activity: ORAC Assay

The antioxidant activity of the MGN-NLC, MGN solution and unloaded NLC was measured using the ORAC assay [53]. Data were obtained using an OPTIMA FLUOstar microplates reader (BMG Labtech, Cary, North California, CA, USA). The assay was conducted at 37 °C, using AAPH (2,2-azobis (2-amidinopropane) dihydrochloride as the oxidant generator, Trolox (0.1 mM) as the control standard and phosphate buffer (pH 7.4) as the blank. A 96-well flat-bottomed black plate was used; the outer rows were filled with 200 μL of distilled water to avoid the evaporation due to the temperature effect. Samples (MGN-NLC, MGN solution and unloaded NLC) were diluted in phosphate buffer (1:1, 1:3, 1:10, 1:20, 1:50, 1:75, 1:100 and 1:150), and then they were deposited (70 μL) in triplicate in the well of the microplate with fluorescein solution (100 μL). Fluorescein solution (3 μM) was prepared in 75 mM phosphate buffer (pH 7.4). Moreover, eight calibration solutions using Trolox (3–100 μM, in phosphate buffer) were added in triplicate (70 μL). After incubation for 15 min at 37 °C, 30 μL of AAPH solution was added, and the fluorescence measurement started. The fluorescence was recorded every 2 min for 180 min (excitation 485 nm, emission 520 nm).

### 4.9. Ocular Tolerability: HET−CAM Assay

Ocular irritancy of unloaded and MGN-loaded NLC were evaluated through the Hen’s Egg Test on Chorio-Allantoic Membrane (HET−CAM), as previously described [18]. The eggshell of incubated (37 °C, 60% relative humidity, 9 days), fertilized hen’s eggs (50–60 g, Coren, Spain) was pierced at the air chamber side using a rotatory saw (Dremel 300, Breda, The Netherlands), and the inner membrane was wet with 0.9% NaCl for 30 min (37 °C). Afterwards, the inner egg membrane was carefully removed to avoid any damage to the fine blood vessels of the chorioallantoic membrane. 

Unloaded NLC and MGN-NLC (100 μL) (in duplicate) were dropped on the CAM. As negative and positive controls, 300 µL of 0.9% NaCl and 0.1 N NaOH solutions were placed on the CAM, respectively. Finally, the irritation score (IS) was calculated as previously reported after observing the CAM for 5 min regarding hemorrhage, vascular lysis or coagulation [35].

### 4.10. Hemolysis Assay

In vitro hemolysis assays were performed for unloaded NLC and MGN-NLC following a slightly modified method used by Chen et al. [54]. A hemolysis assay was performed using freshly drawn whole blood from anonymized healthy donors (Galician Blood Transfusion Center, Santiago de Compostela, Spain) [55,56]. Briefly, blood samples (5 mL) were diluted with 0.9% NaCl (145 mL). Samples (100 μL; dilution 1:5) were added in diluted blood (1 mL) while positive and negative controls were incubated with a Triton X-100 (4%) and a phosphate buffer (PBS commercial, 10×), respectively. After incubation (37 °C) at 100 rpm for 1 h, the samples were centrifuged (Sigma 2-16P; Sigma Laboratory Centrifuges, Germany) at 10,000 rpm for 30 min. The release of hemoglobin was monitored by measuring the absorbance of the supernatant (150 μL) at 540 nm using a microplates reader (FLUOstar Optima, BMG Labtech, Cary, North California, USA). The percentage of hemolysis was calculated as follows Equation (3):

(3) $$\%\;Hemolysis=\frac{\left(A_{S}-A_{N}\right)}{\left(A_{P}-A_{N}\right)}\times 100$$

where $A_{S}$ is the absorbance of sample, $A_{N}$ is the absorbance of negative control and $A_{P}$ is the absorbance of positive control.

### 4.11. Statistical Analysis

Statistical data analysis was performed using Student’s *t*-test followed by a post hoc Bonferroni-Dunn test. A probability (*p*) of less than 0.05 was considered significant.

## 5. Conclusions

MGN has a wide range of pharmacological properties, including anti-inflammatory and antioxidant activities, and can be considered a good candidate for the potential treatment of eye diseases. Its use in ophthalmology is compromised due to its high lipophilicity. This obstacle has been overcome by encapsulating this compound in the NLC. MGN-NLC showed an appropriate particle size, good stability, and high ophthalmic tolerability. The DSC studies indicate that MGN-NLC could enter the biomembrane model and release MGN, and further studies have been planned to prove this hypothesis. Furthermore, the antioxidant activity of MGN-NLC was higher than the free compound. This demonstrated that the carrier preserved the drug. Therefore, these findings suggest that the NLC system is a potential strategy to improve ocular bioavailability of lipophilic drugs.

## Figures and Tables

**Table 1:** The values of size (Z-Ave), PDI and Z-potential for unloaded and MGN-NLC recorded at 25 °C.

Formulation	Z-Ave (nm ± SD)	PDI (–) ± SD	ZP (mV ± SD)
Unloaded NLC	123.1 ± 0.1	0.18 ± 0.10	−28.6 ± 0.3
MGN-NLC	148.9 ± 0.1	0.21 ± 0.02	−23.5 ± 0.2

**Table 2:** Enthalpy variation (ΔH) of MLV left in contact with NLC or MGN-NLC at increasing calorimetric heating scans.

Calorimetric Scan	NLC DH (J/mmol)	MGN-NLC DH (J/mmol)
0	30.30 ± 0.50	30.30 ± 0.50
1	30.30 ± 0.52	29.60 ± 0.51
2	25.35 ± 0.54	23.30 ± 0.72
3	25.20 ± 0.61	23.30 ± 0.73
4	23.19 ± 0.91	22.32 ± 0.64
5	20.07 ± 0.83	20.38 ± 0.80
6	19.88 ± 0.74	20.16 ± 0.82
7	19.65 ± 0.70	19.39 ± 0.90
8	19.51 ± 0.73	18.92 ± 0.72

**Table 3:** The antioxidant activity of unloaded NLC, MGN-NLC and MGN solution. Results are presented as the mean ± S.D., n = 3.

Sample	Trolox-Equivs. (μM TE/g)
Unloaded NLC (1:50)	769 ± 52
MGN-NLC (1:75)	6494 ± 186
MGN solution (1:50)	3521 ± 271

## Data Availability

Data is available on the request from the corresponding author.

## Supplementary Materials

The following supporting information can be downloaded online. Figure S1: Calorimetric curves, in heating mode, of Compritol, Lutrol, Mygliol and MGN.

## Sample Availability

Samples of the MGN, unloaded and MGN-NLC are available from the authors.
