# Supplementary figures and images for: Design of Nanotechnological Carriers for Ocular Delivery of Mangiferin: Preformulation Study
Source: Molecules. 2022 Feb 16;27(4):1328. doi: 10.3390/molecules27041328 (PMC8880740; doi:10.3390/molecules27041328)

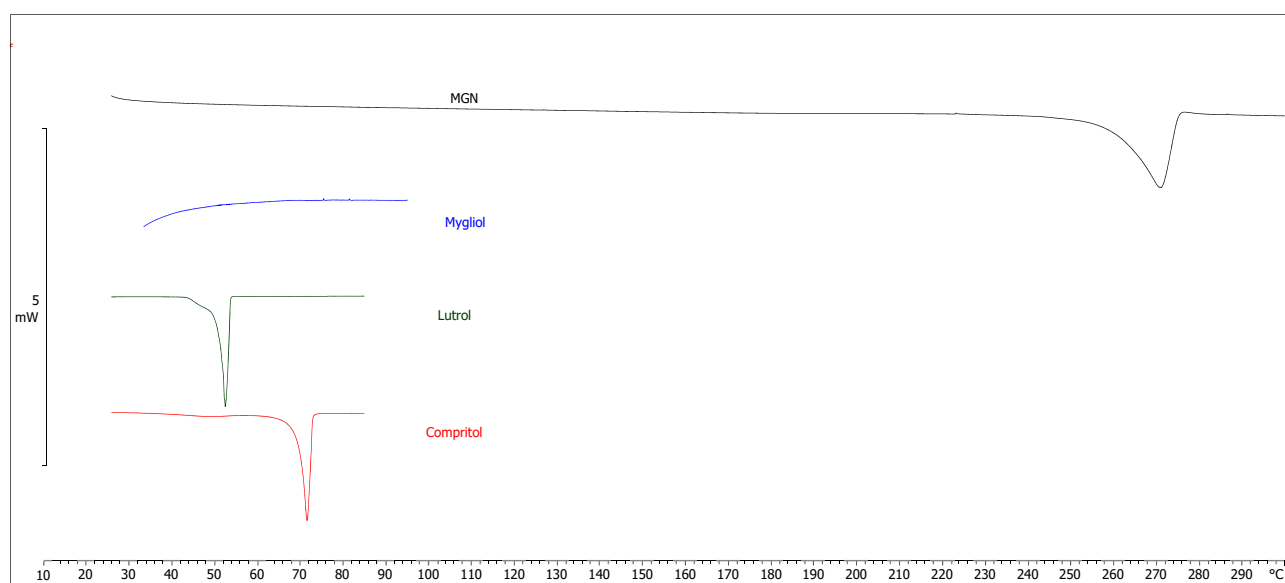

**Figure S1.** Calorimetric curves, in heating mode, of Compritol, Lutrol, Mygliol and MGN.

Supplement: Supplementary file 1 [file molecules-27-01328-s001.zip › molecules-1572360-supplementary.pdf]
